# Comparative assessment of amino acids and volatile compounds role in the formation of wines sensor properties by means of covariation analysis

**DOI:** 10.1016/j.heliyon.2019.e02626

**Published:** 2019-10-11

**Authors:** Z.A. Temerdashev, A.A. Khalafyan, Yu.F. Yakuba

**Affiliations:** a Kuban State University, 149 Stavropol'skaya St., Krasnodar, 350040 Russia; b North Caucasian Federal Research Center of Horticulture, Viticulture, Wine-making, 39, 40-let Pobedy St., Krasnodar, 350901, Russia

**Keywords:** Analytical chemistry, Covariance analysis, Amino acids, Volatile compounds, Sensory analysis

## Abstract

The objects of study were 150 samples of natural dry red and white grape wines of Russian origin, obtained by traditional technologies from European and hybrid grape varieties grown in wineries in Krasnodar Krai in 2010–2016. Natural red (Cabernet, Merlot) and white (Aligote, Riesling, Pinot Noir) (alcohol content of 9–13 % by volume, acidity of 4–7 g/dm3), as well as blend wines based on Cabernet Sauvignon, Merlot and Pinot Noir wines made under experimental conditions were analyzed. Chromatographic and electrophoretic methods were used to determine the content of volatile components and amino acids in the studied samples. A sensory assessment of wine quality was carried out by wine specialists working in the wine industry and having professional experience in the field of sensory analysis. Using statistical modeling we carried out a comparative assessment of the role of amino acids - threonine, proline, arginine and volatile compounds - methanol, acetic acid, furfural in the perception of taste and aromatic properties of wines, a general indicator of which is the average tasting rating. High adequacy of the regression model constructed using covariance analysis indicates that mainly amino acids and volatile compounds determine the sensory properties of wines. The dominant role of amino acids in the perception of taste and aromatic characteristics compared to other wine components is mathematically justified in accordance with the criterion of one-dimensional significance. It has been shown that more than 82% of the sensory characteristics of the analyzed wines group fall on the amino acids and volatile compounds under consideration, and less than 18% - on all the others, including titrated acids, free amino acids, mineral components, phenols, etc.

## 1 Introduction

The International organisation of vine and wine (OIV) guidelines for wine components provide indicators necessary for identifying and assessing the quality of wines (OIV-MA-INT-00-2016, 2016). The sensory properties of wine are the main characteristic of the drink, which determines success with consumers (Jackson, 2008; Ribéreau-Gayon et al., 2006). The principles of sensory assessment of consumer preferences are widely used in the wine industry in all countries of the world (Francis and Williamson, 2015). The value, opportunities and difficulties in clarifying the contribution of chemical compounds to the aroma and taste of wine are described in the review (Francis and Newton, 2005). The article provides an overview of the relationship between the sensory properties of wine and volatile compounds using the results obtained by the method of quantitative gas chromatography-olfactometry (GC/O). But, as it was rightly pointed out by Jackson (2017), the determination of wine quality according to its chemical composition is not entirely objective. Most wine connoisseurs tend to agree that they subjectively like the quality of wine more thanks to the tasting assessment.

The development of modern analytical and sensory methods for determining wine components has expanded the specialists’ capabilities to differentiate their sensory properties, to establish the relationship between the chemical composition and the unique sensory properties specific to different grape varieties and wines. Rapp and Mandery (1986) believe that the possibility to obtain the information related to volatile components determination in wine has made it possible to better understand the difficulty of assessing its organoleptic properties. Sensory properties are formed by both volatile and non-volatile compounds that make up wines (Etiévant, 2017). At the same time, volatile compounds largely form their aromatic qualities, while non-volatile ones, interacting with each other, mainly determine their taste properties due to an ensemble of titrable acids, free amino acids, mineral components and phenolic complex (Versini et al., 2008; Yakuba et al., 2016; Heymann et al., 2014). Titrated acids and acetic acid form an acidic flavor, mineral components and amino acids form the unique taste characteristics along with various phenolic compounds in wine. Proteins and peptides in wine determine the important characteristics of wine quality from aroma and taste fullness to foaming for sparkling wines.

Despite the existence of regulatory documents governing the sensory evaluation of wines, expert methods for determining the quality have certain disadvantages. For example, the board of experts, their number, physiological features at the time of tasting, subjectivity in the perception of the organoleptic wines properties, imbalance of wines, etc. affect the results of tasting. Therefore, the processing of expert estimates includes checking the consistency of expert opinions (or the experts' classification if there is no consistency) and averaging the experts’ opinions within the agreed group. Yakuba et al. (2016) reviewed the methods to assess the consistency of expert assessments by various statistical methods for measuring objects. The appeared methods of statistical analysis not only facilitated the technology of statistical data processing, reducing their labor intensity by dozens of times, but allowed to more effectively and efficiently process expert assessments. Sensory-descriptive analysis of wines quality in combination with one-dimensional or multidimensional statistical analysis is widely used to describe various wines (Noble et al., 1984; Noble and Shannon, 1987; Heymann and Noble, 1987; Koussissi et al., 2002; Kontkanen et al., 2005; Etaio et al., 2008; Esti et al., 2010; Khalafyan et al., 2019). Actively used methods of multivariate analysis - the analysis of variance (ANOVA), the analysis of principal components (PCA), the analysis of correspondence (CA), cluster analysis, regression analysis, logit models (Baker and Ross, 2014; Etaio et al., 2008; Rinaldi and Moio, 2018; Vidal et al., 2018; Jose-Coutinho et al., 2015; Petropoulos et al., 2017; Llobodanin et al., 2014), experimental design (Dooley et al., 2012; Hopfer et al., 2012; Khalafyan et al., 2019; Koak et al., 2010; Vismara et al., 2016), etc., significantly expanded the possibilities to study the factors influencing aromatic and flavoring properties of wines. Moreover, covariance analysis is undeservedly rarely used, despite its methodological significance in identifying complex relationships between the objects characteristics of an arbitrary nature. As follows from the analysis of literature sources, taking into account the component composition in many cases allows implementing a more differentiated approach in assessing their contribution to the formation of their aromatic and taste characteristics. Of the statistical methods, covariance analysis is the most methodologically significant to identify such relationships. The method of covariance analysis will allow building a regression model that can mathematically evaluate the contribution of various wines components to the perception of their taste and aromatic characteristics. In this work, we first studied the possibility of using this method to determine the contribution made by amino acids and volatile compounds to the aroma and taste of natural dry red and white grape wines and to establish the relationship between their sensory properties and the contents of these components in the studied samples.

## 2 Materials and methods

***The research objects*** were 150 samples of natural dry red (Cabernet, Merlot) and white (Aligote, Riesling, Pinot Noir) Russian-made grape wines obtained by traditional technologies from European and hybrid grape varieties by industrial producers “Myskhako”, “Fanagoria”, “Kubanvino”, “UVK”, “Villa Victoria”, “Chateau Taman”, “Chateau la Grand Vostok”, as well as blends based on dry wines and made in experimental conditions “Cabernet Sauvignon”, “Merlot” and “Pinot Noir”. The wines were produced in 2010–2013 (alcohol content - 9–13% by volume, acidity - 4–7 g/dm^3^).

For chromatographic and electrophoretic studies, analytical standards of proline, arginine, serine, valine, glycine and threonine, 18-crown ether-6, purchased from Sigma Aldrich (Santa Ana, CA, USA) were used. Cyclodextrin, sodium hydrogen phosphate, potassium dihydrogen phosphate, phenylisothiocyanate, sodium carbonate were of analytical purity and were purchased from Merck (Germany), and b-cyclodextrin, tartaric acid, H_3_PO_4_, HCl, NaOH, Na_2_B_4_O_7_x10H_2_O were purchased from Vekton (Russia).

A stock solution of each analyte (100 mg/L in double-distilled water) was prepared daily, stored at 4 °C and diluted with double-distilled water.

### 2.1 Research methods

The system of capillary electrophoresis of the CAPEL series (Lumex, Russia) with the photometric detector (254 nm); the quartz capillar with an external polyimide coating (bore diameter of 75·10^−6^ m, effective length of 0.5 m; aqueous thermostatic control) were used. At the beginning of each day the capillary was conditioned by flushing with 1 mol/L NaOH (3 min) followed by a 5 min flush with dist water and the elecrtolyte (3 min). In between runs the capillary was reconditioned with the elecrtolyte (2 min flush). At the end of each working day the capillary was rinsed with 1 mol/L NaOH (5 min) and water (5 min). Standard solutions and samples were introduced at the extremity of the capillary nearest the detector and injected hydrodynamically (at 30 mbar for 5 s). The applied separation voltage was 15 kV with positive polarity at the injection end.

Mass concentration of volatile components wasdetermined by the method of capillary gas chromatography with flame ionization detector (GC-FID). GC-FID method was implemented on the gas chromatograph Crystall-2000М (Chromatec, Russia) equipped with the capillary column HP-FFAP with 50 m length, 0.32 mm bore diameter and 0.52 microns film thickness (Agilent, USA) (Yakuba and Temerdashev, 2015). The conditions of the analysis were the following: injector temperature was 200°С; FID detector temperature was 220°С; gas-carrier (nitrogen) flow through a column was 1.21 cm^3^/min; injector split ratio was 1:33; initial oven temperature was 70°С with isotherm of 7 min, further 5 °C/min to 140°С, 10 min plateau, further 10 °C/min to 180°С and endurance till the end of the analysis; the injection volume was 1 mm^3^; the hydrogen flow was 20 cm^3^/min; the air flow was 200 cm^3^/min; the analysis time was 40 min. Quantitative determination was performed by the method of absolute calibration with the model solutions.

Sensory assessment of wines quality was carried out by the specialists of the federal state budgetary scientific institution “North-Caucasian Federal Scientific Center for Horticulture, Viticulture, and Wine-Making” (Krasnodar). All the participants are considered to be experts in the field of wine, they work in the wine industry and have professional experience in the field of sensory analysis. The results of sensory evaluation were expressed on a 100-point rating scale (Parker, 2003). Statistical modeling of the relationship between the sensory properties of wines - tasting assessment and the quality of wines determined by the content of free amino acids and volatile substances, as well as the analysis of their influence degree on aroma and taste was carried out in the medium of STATISTICA (v.10, Tibco) package (Hill and Lewicki, 2007).

## 3 Results and discussion

As a result of chromatographic and electrophoretic studies in the given wine samples, the following amino acids were identified (arginine, proline, threonine, β-phenylalanine, tyrosine, tryptophan), higher alcohols (2-propanol, 1-propanol, 2-butanol, isobutanol, 1-butanol, isoamylol, 1-amylol, 1-hexanol), esters (methyl acetate, ethyl acetate, isoamyl acetate, ethylcaprylate, ethyl lactate), acetaldehyde, methanol, glycerin, tartaric acid, malic acid, lactic acid, citric acid, succinic acid, acetic acid. The level of these components content in wines is different; for more details, see our review article (Yakuba et al., 2014).

Taking into account the data analysis from the literature (Yakuba et al., 2014, 2016; Khalafyan et al., 2016a; Gómez García-Carpintero et al., 2012; Sanchez-Palomo et al., 2019; Sánchez-Palomo et al., 2018; Holmberg, 2010; Lecat et al., 2017) and the obtained experimental material as substances involved in the formation of the aromatic part of the spectrum of wines sensory properties, the data on volatile components were determined and used – acetaldehyde, ethyl acetate, methanol, the total content of higher alcohols, acetic acid, furfural. The choice of these wines components is determined by the fact that they mainly characterize the stages of the technological process and the level of production. From the above mentioned volatile compounds we isolated 3 substances – methanol, acetic acid, and furfural, which affect the quality of wines to a large extent (Khalafyan et al., 2016b). On the basis of the conducted studies, the empirical relationships of volatile compounds concentrations and the quality of natural Kuban wines were proposed, which allowed to classify them into 3 groups – low, medium and high quality:

– if furfural content in the wine sample is above 9 mg/dm^3^, the wine is of poor quality;
– if furfural content in the wine sample is not more than 9 mg/dm^3^, acetic acid and methanol is more than 430 and 95 mg/dm^3^ respectively, the wine is also of poor quality;
– if furfural content in the wine sample is not more than 9 mg/dm^3^, acetic acid is more than 430 mg/dm^3^, and the methanol concentration in the wine sample does not exceed 95 mg/dm^3^, the wine is of average quality;
– if furfural content and acetic acid in the wine sample do not exceed 9 and 430 mg/dm^3^ respectively, and the higher alcohols are more than 150 mg/dm^3^, the wine is of high quality.

**Fig. 1:**
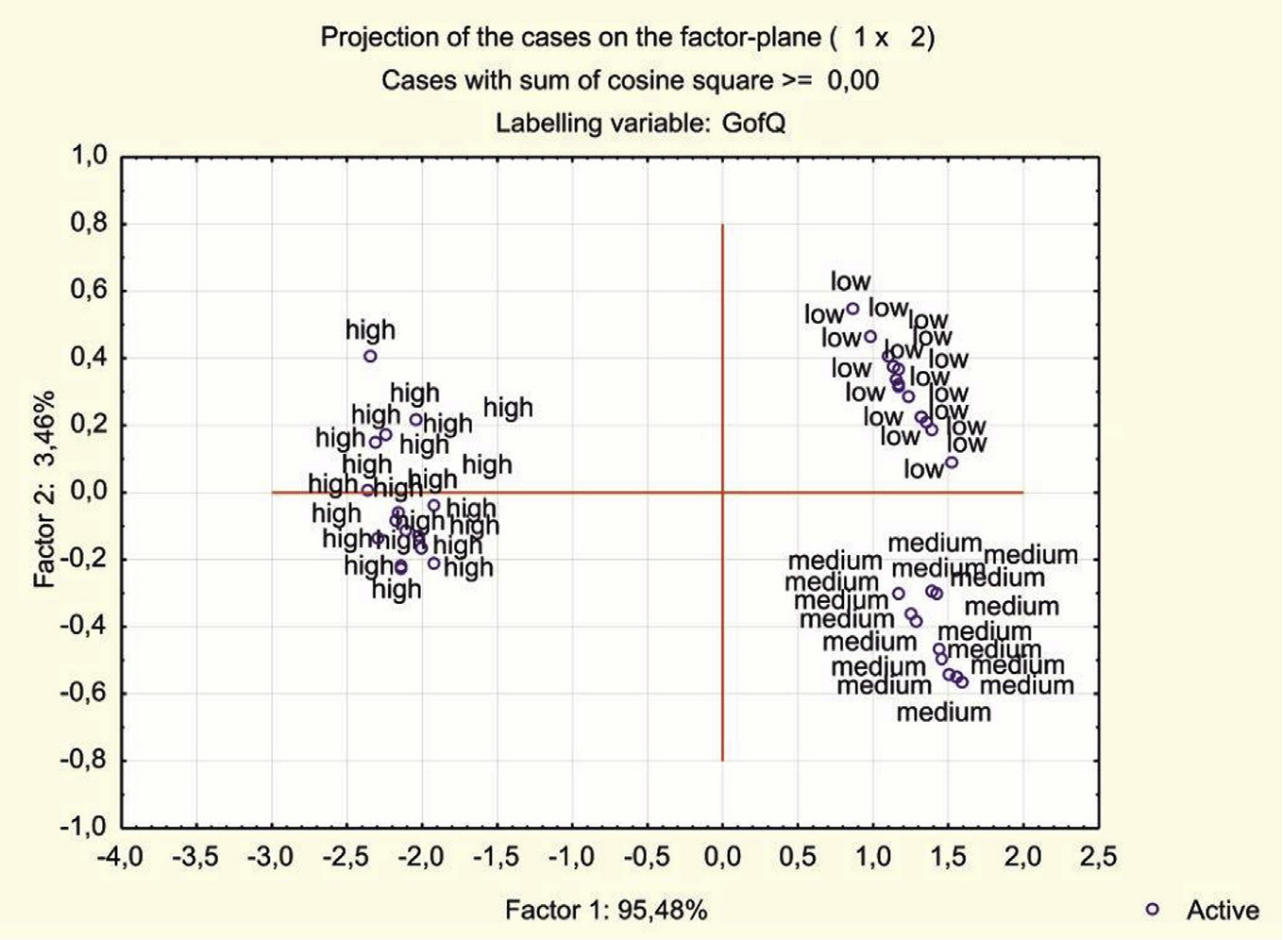
Projection of wines on the factor plane constructed by method PCA.

Taking into account the established empirical relationships, the tested 150 wine samples were divided into 3 groups each containing 50 samples of high, medium and low quality with average values of sensory assessments equal to 81.76; 72.14 and 68.72 respectively. The fact that such a division determines homogeneity groups follows from the statistical significance of the differences in mean values in accordance with the Kruskal-Wallis criterion. The graphic confirmation is the graph of wines projection on the factor plane constructed by the method of *Principal Component Analysis and Classification* (PCA) (Fig. 1).

As can be seen from the Figure, the wines of 3 quality groups are localized in different parts of the plane at a considerable distance from each other, and the wines of average quality are located between the wines of high and low quality. From the aggregate of the analyzed amino acids, 3 amino acids were identified by the correlation analysis – arginine, proline, threonine, which are most closely related to the taste component of the wine tasting assessment. The degree of interrelation of wines sensory properties with the concentrations of amino acids and quality groups was assessed using the Spearman's rank correlation coefficient (r). For a shorter further recording, the following notation is introduced: tasting evaluation – Est; the amino acid concentrations of proline, threonine and arginine are respectively *C*_*Pr*_*, С*_*Trn*_*, С*_*Arg*_; quality group – *QGrp*. The strongest correlation (|r|> 0.75) is observed in the groups of volatile substances (|r| = 0.845) and *С*_*Pr*_ level (r = 0.844), the third place is taken by *С*_*Arg*_ (r = 0.627) and the fourth – *С*_*Trn*_ (r = 0.523) with moderate correlations (0.25 <|r| <0.75). The given statistically significant values of the correlation coefficients characterize the degree of interrelation without considering the joint influence of volatile substances and amino acids on the sensory properties of wines.

The presence of strong and moderate correlations for quantitative indicators is a prerequisite for the construction of a regression equation that models the relationship between the response – the tasting assessment and the totality of predictors – the concentrations of amino acids. This equation along with the predictive capabilities will allow evaluating the relationship of the response with the ensemble of predictors and the contribution of each predictor to the regression model. Table 1 presents the results of the regression analysis obtained by the *Multiple Regression* module of the STATISTICA package. The information part of Table 1 shows the values of the multiple correlation coefficient R = 0.9 and the coefficient of determination R^2^ = 0.807, characterizing the correlation of predictors in the aggregate and the response, as well as the variability of the response described by the model relative to the average value. R and R^2^ values close to 1 indicate a strong correlation between the response and predictors, as well as a high adequacy of the regression model.

**Table 1:** Results of multiple regression analysis characterizing the parameters of the regression model.

N = 150	Regression Summary for Dependent Variable: Est R = 0.901; R^2^ = 0.811; Adjusted R^2^ = 0.807 F(3.146) = 208.75 p < 0.000; Std.Error of estimate: 2.755					
	b*	Std.Err. of b*	b	Std.Err. of b	t(146)	p-value
Intercept			**67.790**	**0.355**	**191.220**	**0.000**
*С*_*Arg*_	0.022	0.129	0.014	0.082	0.172	0.864
***С***_***Pr***_	**0.991**	**0.093**	**0.023**	**0.002**	**10.673**	**0.000**
*С*_*Trn*_	– 0.129	0.106	– 0.041	0.034	–1.220	0.224

The b * column shows the coefficients of the standardized equation, whose values characterize the predictors contribution to the model. As can be seen, the greatest contribution among them is made by *С*_*Pr*_, the smallest one – by *С*_*Arg*_. In the regression model the free member and *C*_*Pr*_ (the corresponding lines are shown in bold italics) are statistically significant, as evidenced by their significance levels of the p t - criterion (Student), which are below 0.05. Taking into account the model parameters given in column b, the multiple linear regression equation has the form:

(1) *Est* = 67.790 + 0.014·*С*_*Arg*_ + 0.023·*С*_*Pr*_ – 0.041·*С*_*Trn*_

The regression Eq. (1) is statistically significant, since the significance level p of the F-criterion (Fisher) is less than 0.05, which also indicates the adequacy of the constructed model.

The presence of a strong correlation for the qualitative (categorical) predictor of *QGrp* is a prerequisite to a statistically significant difference in the average values of tasting evaluation in groups of quality – *high, medium, low*. The analysis of variance confirmed the statistically significant difference between the average values of the tasting assessment in the indicated groups – all the levels of criterion significance the least significant difference (LSD) were less than 0.05. Thus, the arithmetic average in the group is high (81.760), which is statistically significantly higher than the arithmetic average in the group of *medium* (72.14) and *low* (68.70); in its turn, the arithmetic average in the group *medium* (72.14) is statistically significantly higher than the arithmetic average in the *low* group (68.70).

Using a one-dimensional significance criterion, the analysis of variance (ANOVA) allows us to estimate the variability of the response – the *Est* tasting assessment. The statistics characterizing the variability of *Est* are given in Table 2, where SS is the sum of squared deviations or variability determined by the difference in mean values between groups; *MS* is the average sum of squared deviations, which is calculated as the ratio of *SS* to the number of freedom degrees. The higher the value of these statistics, the greater the variability of the response is determined by the categorical predictor.

**Table 2:** One-dimensional criterion for the significance of variance analysis, which characterizes the variability of the sensory assessment of wine quality.

Effect	Univariate Results for Each DV Sigma-restricted parameterization Effective hypothesis decomposition				
	Est SS	Degr. of Freedom	Est MS	Est F	Est p
Intercept	825994.4	1	825994.4	94036.01	0.00
*QGrp*	4571.4	2	2285.7	260.22	0.00
Error	1291.2	147	8.8		
Total	5862.6	149			

Covariance analysis is, in fact, a synthesis of regression and variance analysis, as it allows investigating the nature of the relationship between response and a set of quantitative and qualitative predictors. Quantitative predictors in covariance analysis are called covariates. Covariates and categorical predictors, as in the analysis of variance, are called effects.

While the variance analysis assesses the degree of random variability of the response from the side of the effects – categorical predictors and their combinations, in the covariance analysis the degree of response variability is evaluated from the side of quantitative predictors – the covariate. Concerning covariates, assumptions are made that, along with qualitative effects, they cause a certain amount of response variability. If the degree of response variability from covariates is large, we talk about a statistically significant effect of covariates on the response. At the same time, the key point of the covariance analysis is that it allows to estimate the degree of covariates influence on the impact of the categorical predictor on the response. In the analysis of experimental data under consideration, the influence of amino acids concentration on the effect exerted by the quality group, determined by the concentration of volatile substances, on the value of the tasting evaluation is assessed.

Table 3, constructed using the covariance analysis, presents the parameters of a one-dimensional significance criterion, from which it follows that the *C*_*PR*_ is the largest contributor to the regression model, and therefore to the joint description of wines quality, since SS takes the maximum value (211.223). Next are the effects: the quality group *– QGrp, С*_*Trn*_ and *С*_*Arg*_. At the same time, all the effects are statistically significant in the model, with the exception of *С*_*Arg*_ (the significance p level of the F-criterion given in the last column of the table is significantly greater than 0.05).

**Table 3:** One-dimensional criterion of the covariance analysis significance characterizing the predictor contributions to the regression model.

Effect	Univariate Results for Each DV Sigma-restricted parameterization Effective hypothesis decomposition				
	Est SS	Degr. of Freedom	Est MS	Est F	Est p
Intercept	**3446.436**	**1**	**3446.436**	**472/938**	**0.000**
*С*_*Arg*_	0.190	1	0.190	0.026	0.871
***С***_***Pr***_	**211.223**	**1**	**211.223**	**28.985**	**0.000**
***С***_***Trn***_	**31.954**	**1**	**31.954**	**4.384**	**0.038**
*QGrp*	**59.012**	**2**	**29.506**	**4.049**	**0.019**
Error	1049.368	144	7.287		
Total	5862.593	149			

Taking into account the values of the regression model coefficients given in Table 4, the regression equation constructed by the covariance analysis takes the form:

(2) *Est* = 64.482–0.017·*С*_*Arg*_ + 0.039·*С*_*Pr*_ – 0.101·С_Trn_ – 3.906·*QGrp*_*1*_+0.024·*QGrp*_*2*_

**Table 4:** The parameters of the regression model of covariance analysis for sensory Est evaluation.

Effect	Parameter Estimates Sigma-restricted parameterization					
	Level of Effect	Column	Est Param.	Est Std.Err	Est t	Est p
Intercept		**1**	**64.482**	**2.965**	**21.747**	**0.000**
*С*_*Arg*_		2	–0.017	0.104	–0.161	0.871
***С***_***Pr***_		**3**	**0.039**	**0.007**	**5.383**	**0.000**
***С***_***Trn***_		**4**	–**0.101**	**0.048**	–**2.094**	**0.038**
*QGrp*_*1*_	high	5	–3.906	3.451	–1.131	0.259
*QGrp*_*2*_	medium	6	0.024	1.470	0.016	0.986

Guided by the principle of sigma-limited parametrization, the categorical predictor takes only 2 values for coding in the binary system with the values 0 and 1. Therefore, the *QGrp* predictor which takes 3 values is represented as two predictors *QGrp*_*1*_ and *QGrp*_*2*_ with two values each – *high, low and medium, low*. From the last column of Table 4 it is seen that in Eq. (2) the predictors of *С*_*Pr*_, *С*_*Trn*_ and the free term are statistically significant – the significance levels of the p t-test are less than 0.05.

It follows from Table 5 that the constructed model (2) of the relationship of tasting evaluation (Est) with predictors – concentrations of amino acids and quality groups is sufficiently adequate, since the coefficient of multiple correlation R = 0.906 is close to 1. The coefficient of determination R^2^ = SS_Model_*/(*SS_Model_ *+* SS_remainder_) = 0.821 describes more than 82% of the response variability relative to the mean value. This means that more than 82% of the organoleptic characteristics in the analyzed group of wines fall on the concentrations of amino acids and volatile compounds considered as predictors, and less than 18% – on all the other components, including titrated acids, free amino acids, mineral constituents, phenols, etc.

**Table 5:** Parameters characterizing the adequacy of the regression model.

Dependent Variable	Test of SS Whole Model vs. SS Residual							
	Multiple R	Multiple R^2^	SS Model	MS Model	SS Residual	MS Residual	F	p
*Est*	0.906	0.821	4813.221	962.645	1049.368	7.287	132.0	0.00

Using Table 6, you can set the categorical predictor coding rule in the regression equation based on the sigma-limited parameterization principle – the values of the predictors *QGrp*_*1*_ and *QGrp*_*2*_ recorded in the Level of Variable column are encoded 1, in the Versus Level column – 0.

**Table 6:** Marks of regression equation columns setting the rule for categorical predictor coding.

Label	Column Labels Labels for the columns of the design matrix X			
	Column	Variable	Level of Variable	Versus Level
Intercept	1			
*С*_*Arg*_	2	*С*_*Arg*_		
*С*_*Pr*_	3	*С*_*Pr*_		
*С*_*Trn*_	4	*С*_*Trn*_		
*GofQ*_*1*_	5	*GofQ*_*1*_	high	low
*GofQ*_*2*_	6	*GofQ*_*2*_	medium	low

We illustrate the calculation using Eq. (2) on the example of a wine sample with a well-known tasting score 84, belonging to the *high* quality group if *С*_*Arg*_ = 25, *С*_*Pr*_ = 654, *С*_*Trn*_ = 54:

(3) Est = 64.482–0.017 25 + 0.039 · 654–0.101 54–3.906 1 + 0.024·0 = 80.203

It is easy to calculate that the forecast error was 4.5% of the original estimate of 84, i.e. we got a fairly accurate forecast. Consequently, it was possible to predict the value of the tasting assessment and build a completely adequate model based on 3 amino acid concentrations – *proline, threonine, arginine* and a *high* quality group, whose membership is determined by the concentrations of methanol, acetic acid, furfural. The model can be used to predict the tasting assessment by the results of chemical analysis of wines.

But we can get the same result using the method of *General linear models* (Khalafyan et al., 2016a)! The advantage of the covariance analysis is that it allows an assessment of the predictors contribution to the variability of the response. Thus, according to the results of the covariance analysis, it can be argued that the variability of the response – *the tasting assessment* is influenced by the covariates of amino acids and the quality group. In this case, from Table 3 it follows that *С*_*Pr*_ covariates have the largest contribution (SS = 211.223), which is more than 3 times more than the contribution of the *quality group* (SS = 59.012). It is important to know that after the introduction of covariates into the model of the interrelation between the *quality group* and *tasting assessment*, the contribution of the quality group which includes methanol, acetic acid, furfural decreased almost 77 times from 4571.4 (Table 2) to 59.012 (Table 3). At the same time, despite the dominant effect of covariates on the response, the impact of the *quality group*, and hence volatile compounds on the response, still retained statistical significance - the *p* significance level of the *F-criterion* for the predictor *QGrp* = 0.019, less than 0.05 (Table 3).

## 4 Conclusions

The set of amino acids considered in the paper – threonine, proline, arginine and volatile compounds – methanol, acetic acid, furfural sufficiently determines the sensory properties of wines, which follows from the high adequacy of the regression model constructed by the covariance analysis. At the same time, it should be noted (which is mathematically quite reasonable) in accordance with the criterion of one-dimensional significance that the role of amino acids is more important than the role of volatile compounds in the perception of taste and aroma characteristics of wines by experts, the consolidated indicator of which is the results of tasting. Moreover, more than 82% of the sensory characteristics of the analyzed group of wines fall on the considered amino acids – proline, threonine, arginine and volatile compounds – methanol, acetic acid, furfural, less than 18% – on all the other components, including titrated acids, free amino acids, mineral components, phenols, etc.

## Declarations

### Author contribution statement

Zaual Temerdashev: Conceived and designed the experiments; Analyzed and interpreted the data; Contributed reagents, materials, analysis tools or data; Wrote the paper.

Aleksan Khalafyan: Conceived and designed the experiments; Performed the experiments.

Yuri Yakuba: Performed the experiments; Contributed reagents, materials, analysis tools or data.

### Funding statement

This work was supported by the Ministry of Education and Science of the Russian Federation, project no. 4.2612.2017/PCh and the Russian Foundation for Basic Research (grant no. 18-03-00059).

### Competing interest statement

The authors declare no conflict of interest.

### Additional information

No additional information is available for this paper.
